# Letter to the editor: Addressing radiological terminology of basal ganglia and thalamic injury in hypoxic ischaemic injury

**DOI:** 10.4102/sajr.v25i1.2146

**Published:** 2021-05-26

**Authors:** Bates Alheit

**Affiliations:** 1 Private Radiology, Cape Town, South Africa

I refer to a recent webinar presented by Prof. Savvas Andronikou on the patterns of hypoxic ischaemic brain injury (HIBI) in children when scanned by magnetic resonance (MR) at an older age.

I write this letter in support of Prof. Andronikou’s suggested approach to the basal ganglia and thalamus (BGT) pattern of injury and the conditions under which the terminology of an ‘acute profound’ injury should be used.

I refer to the pictorial review by Misser et al. on the correlation of the pathophysiology and magnetic resonance imaging (MRI) patterns in children with HIBI that appeared in the *South African Journal of Radiology (SAJR)* in October 2020.^1^ In this article, the authors classify MRI patterns and ascribe pathogenetic mechanisms to each. They refer to classic bilateral hypoxic ischaemic insults of the posterior putamina and ventrolateral thalami as an ‘acute profound pattern’^1^ and specify that the description of acute profound injury links this particular diagnosis with sentinel events, which include ruptured uterus, abruption of the placenta and prolapsed cord.^2,3,4^ A large number of cases with the BGT injury pattern have no record of having suffered a sentinel event; that is, naming these as an acute profound hypoxic ischaemic injury is inappropriate.

I draw the attention of the readership to the evolution of this particular terminology, which has come about in an effort to just avoid such pathogenetic assumptions associated with the involvement of the deep nuclei on MRI. Although it is widely accepted that watershed injuries occur as a result of partial and prolonged (usually over 1 h) hypoxic ischaemic injury to the term foetus,^5,6^ injury to the BGT has been shown to also occur without any sentinel event having occurred.^7^ Hence, the currently preferred term ‘BGT pattern of injury’ has emerged in publications dating from as far back as 2011, which does not contain any reference to the duration or severity of the injury.^8,9,10,11,12^

A recent article by Smith et al.^7^ specifically addresses this issue through a patient cohort carefully selected for good clinical documentation, who had an MRI pattern of ‘BGT pattern of injury’, but did not have any sentinel event or clinical *acute profound HIBI*. The authors concluded therefore that the terms ‘acute profound pattern’ and ‘BGT pattern’ are not synonymous. These authors demonstrated that a *prolonged non-reassuring foetal status* (NRFS) during labour, leading to a pathological tracing in the absence of a perinatal sentinel event, can result in a ‘BGT pattern of injury’ on MRI. These cases should not erroneously be referred to as having an ‘acute profound injury’.

A number of other scientific publications support the findings of Smith et al. Harteman et al. reported that 11 of the 18 cases with ‘BGT pattern of injury’ did not have sentinel events. All had abnormal foetal heart rate (FHR) patterns before birth, suggesting that injury to the deep nuclei was most probably because of foetal distress.^10^ Bonifacio et al. reported that a perinatal sentinel event was present in only 10 of the 60 hypoxic ischeamic encephalopathy (HIE) cases, but that 14 cases overall had a ‘BGT pattern of injury’ as the predominant pattern on MRI – therefore, four cases of BGT injury (28.5%) did not have a sentinel event.^11^ Barnette et al. reported 36 cases of HIE with a ‘BGT pattern of injury’ without a perinatal sentinel event or shoulder dystocia.^13^ Miller et al. demonstrated that foetal distress was the probable cause of HIE in 23 of the 44 babies with BGT predominant injury.^14^ Naeye et al. reported *two cases of BGT injury that were clearly not related to a sentinel event –* one had a bradycardia for 2 h before delivery and the other had cessation of foetal movements for 28 h before birth.^15^ Martinez-Biarge et al. reported that *at least 230 (59.5%) of the 393 cases with* ‘acute hypoxia ischaemia’ on MRI had no sentinel event. An abnormal cardiotocograph (CTG) was present in at least 276 of the total cohort, which strongly suggests that foetal distress was the cause for acute hypoxic injury in as many as 113 (276−163 = 113) cases (28%).^16^

Why is it important for us NOT to report the involvement of the deep nuclei in perinatal hypoxic ischaemic as an ‘acute profound’ pattern? Smith et al. draw attention to a medicolegal lexicon, which has evolved in South Africa specifically, where it is implied that ‘acute profound HIBI’ is ‘always’ sudden (acute) and ‘always’ profound (severe and total). This view has fostered the belief in the courts that very little could have ‘ever’ been done to arrest the process of foetal neurological injury where that injury is reported as ‘acute profound’ on MRI.

In the light of the above, the author of this letter recommends that abnormalities of the posterior putamina and ventrolateral thalami, as described by Misser et al. in term perinatal hypoxic ischaemic injury, should be reported as a ‘BGT pattern of injury’ in the absence of a documented sentinel event, as was elegantly explained in the seminar alluded to above. If there is no definitive confirmation of a preceding sentinel event, a radiologist is not in a position to deduce from the structural damage identified on MR images under what clinical or obstetrical conditions this type of injury occurred. It is also noted that the use of the term ‘acute profound’ remains valid, provided it is used in accordance with the ACOG 2014 definition of an acute profound injury and that there is definitive obstetrical evidence of a sentinel event that preceded the insult.^17^

So what can the radiologist say about the timing of the injury? Some indications that the injury occurred in a brain of ‘term maturity’ are provided by the presence of glial strands in multicystic encephalomalacia,^9,18,19^ ulegyria in watershed injury^20,21^ and involvement of the peri-Rolandic region,^5,21^ but radiologists are not able to determine the exact timing of the injury during the peri-partum period. In this context, it is notable that the vast majority of hypoxic ischaemic injuries occur during the peri-partum period.^9,22^

Radiologists should defer to clinical and obstetrical experts to advise on the clinical context, the probable causation, timing and severity of the insult.

Dr B. Alheit

MB. ChB. MMed Diag. Radiology (Stellenbosch)

## Notes

Response to Letter to Editor

Dear Editor,

Your request for a response to the letter to editor by Dr Alheit, dated March 2021, refers.

Challenges to the existing theories of perinatal hypoxic ischaemic brain injury (HIBI) are ongoing and continue to contribute to the body of knowledge in this sphere of paediatric neuroradiology. We have tried to address Dr Alheit’s query; however, we feel that the scope and intent of our publication have been misunderstood. For the purpose of clarity, the reader is referred to the abstract and conclusion of the article. The cases in our article^1^ that we described as acute profound HIBI were children who had suffered sentinel events, examples of which we cited as abruptio placentae, cord prolapse or uterine rupture. These clinical factors were clearly described in our article along with the relevant pathophysiological processes.

Nomenclature and classifications are increasingly being referenced in medicolegal matters in South Africa. The classification we have described has been widely accepted and in use up to the time of publishing. Further support was confirmed through article review by peer-review process. Our purpose, as radiologists, is to describe the pattern we see on imaging and not be drawn into a debate on medicolegal terminology. Up until October 2020, when our article was published, radiological reporting of the central pattern of injury (including basal ganglia, thalamus and rolandic injury), which we have called acute profound injury, has previously been ascribed to an acute pathophysiology. Cognisance is taken of the recent article quoted,^2^ showing that a basal ganglia thalamus (BGT) pattern may also occur in the absence of sentinel events and is therefore not synonymous with acute profound HIBI. We are not opposed to the use of the anatomical description proposed, to call this injury a BGT pattern, where there is no correlative sentinel event documented. Structured research studies interrogating these propositions are encouraged.

The fact is that the full extent of any perinatal event is not fully understood. The patterns that have been described (by my group and others) are most certainly real, but a close monitoring of ongoing physiological changes in human babies during the delivery is rare, especially in resource-constrained settings. We can only surmise what has happened later, after the child has been delivered. Physicians make observations, based upon the information that they obtain before, during and after delivery and then work retrospectively to try to understand what happened.

Frankly, NOBODY completely understands all of the processes that take place in these cases. A very important concept is that every HIBI episode is different. Indeed, it is likely that every one of them is a little different from all the others. Added to that is the fact that each body probably reacts a little differently to an ischaemic insult. When one considers all the different physiological processes ongoing in these babies, and the fact that everyone’s physiology is slightly different, it is not surprising that the pattern of injury in each one is a little different from all the others. In science, one cannot be too dogmatic when describing expected findings. Everyone has a slightly different physiology and is going to have slightly different responses to an event. However, our findings indicate that what we have published is true for a large (perhaps even a vast) majority of affected patients.

## Notes

Dr Shalendra K. Misser (Corresponding author)

Radiologist, Lakesmit and Partners Inc, Durban, South Africa

## References

[1] Misser SK, Barkovich AJ, Lotz JW, Archary M. A pictorial review of the pathophysiology and classification of the magnetic resonance imaging patterns of perinatal term hypoxic ischemic brain injury – What the radiologist needs to know…. SA J Radiol. 2020 10 30;24(1):1915. 10.4102/sajr.v24i1.1915 33240541 PMC7670012

[2] De Vries LS, Groenendaal F. Patterns of neonatal hypoxic-ischaemic brain injury. Neuroradiology. 2010 6;52(6):555–566. 10.1007/s00234-010-0674-9 20390260 PMC2872019

[3] Rutherford M, Srinivasan L, Dyet L, et al. Magnetic resonance imaging in perinatal brain injury: Clinical presentation, lesions and outcome. Pediatr Radiol. 2006 7;36(7):582–592. 10.1007/s00247-006-0164-8 16770663

[4] Rutherford M, Malamateniou C, McGuinness A, Allsop J, Biarge MM, Counsell S. Magnetic resonance imaging in hypoxic-ischaemic encephalopathy. Early Hum Dev. 2010 6 12;86(6):351–360. 10.1016/j.earlhumdev.2010.05.014 20541877

[5] Reid SM, Dagia CD, Ditchfield MR, Reddihough DS. Grey matter injury patterns in cerebral palsy: Associations between structural involvement on MRI and clinical outcomes. Dev Med Child Neurol. 2015 12;57(12):1159–1167. 10.1111/dmcn.12800 25970144

[6] Robertson CM, Perlman M. Follow-up of the term infant after hypoxic-ischemic encephalopathy. Paediatr Child Health. 2006 5;11(5):278–282. 19030289 PMC2518676

[7] Smith J, Solomons R, Vollmer L, et al. Intrapartum basal ganglia-thalamic pattern injury and radiologically termed ‘acute profound hypoxic-ischemic brain injury’ are not synonymous. Am J Perinatol. In press 2020 12 15. 10.1055/s-0040-1721692 33321532

[8] Hayakawa K, Tanda K, Koshino S, Nishimura A, Kizaki Z, Ohno K. Pontine and cerebellar injury in neonatal hypoxic-ischemic encephalopathy: MRI features and clinical outcomes. Acta Radiol. 2020 10;61(10):1398–1405. 31979976 10.1177/0284185119900442

[9] Ghei SK, Zan E, Nathan JE, et al. MR imaging of hypoxic-ischemic injury in term neonates: Pearls and pitfalls. Radiographics. 2014 8;34(4):1047–1061. 10.1148/rg.344130080 25019441

[10] Harteman JC, Nikkels PGJ, Benders MJNL, Kwee A, Groenendaal F, De Vries LS. Placental pathology in full-term infants with hypoxic-ischemic neonatal encephalopathy and association with magnetic resonance imaging pattern of brain injury. J Pediatr. 2013 10;163(4):968.e2–995.e2. 10.1016/j.jpeds.2013.06.010 23891350

[11] Bonifacio SL, Glass HC, Vanderpluym J, et al. Perinatal events and early magnetic resonance imaging in therapeutic hypothermia. J Pediatr. 2011 3;158(3):360–365. 20965514 10.1016/j.jpeds.2010.09.003 PMC3035732

[12] Lakatos A, Kolossváry M, Szabó M, et al. Neurodevelopmental effect of intracranial hemorrhage observed in hypoxic ischemic brain injury in hypothermia-treated asphyxiated neonates – An MRI study. BMC Pediatr. 2019 11 12;19(1):430. 10.1186/s12887-019-1777-z 31718607 PMC6849254

[13] Barnette AR, Horbar JD, Soll RF, et al. Neuroimaging in the evaluation of neonatal encephalopathy. Pediatrics. 2014 6;133(6):e1508–e1517. 24864165 10.1542/peds.2013-4247

[14] Miller SP, Ramaswamy V, Michelson D, et al. Patterns of brain injury in term neonatal encephalopathy. J Pediatr. 2005 4;146(4):453–460. 10.1016/j.jpeds.2004.12.026 15812446

[15] Naeye RL, Lin HM. Determination of the timing of fetal brain damage from hypoxemia-ischemia. Am J Obstet Gynecol. 2001 1;184(2):217–224. 10.1067/mob.2001.108996 11174505

[16] Martinez-Biarge M, Diez-Sebastian J, Wusthoff CJ, Mercuri E, Cowan FM. Antepartum and intrapartum factors preceding neonatal hypoxic-ischemic encephalopathy. Pediatrics. 2013 10;132(4):e952–e959. 10.1542/peds.2013-0511 24019409

[17] Executive summary: Neonatal encephalopathy and neurologic outcome, second edition. Report of the American College of Obstetricians and Gynecologists’ Task Force on Neonatal Encephalopathy. Obstet Gynecol. 2014 4;123(4):896–901. 24785633 10.1097/01.AOG.0000445580.65983.d2

[18] Barkovich AJ, Truwit CL. Brain damage from perinatal asphyxia: Correlation of MR findings with gestational age. AJNR Am J Neuroradiol. 1990 12;11(6):1087–1096. 2124034 PMC8332119

[19] Counsell SJ, Tranter SL, Rutherford MA. Magnetic resonance imaging of brain injury in the high-risk term infant. Semin Perinatol. 2010 2;34(1):67–78. 10.1053/j.semperi.2009.10.007 20109974

[20] Nikas I, Dermentzoglou V, Theofanopoulou M, Theodoropoulos V. Parasagittal lesions and ulegyria in hypoxic-ischemic encephalopathy: Neuroimaging findings and review of the pathogenesis. J Child Neurol. 2008 1;23(1):51–58. 10.1177/0883073807308694 18160553

[21] Shroff MM, Soares-Fernandes JP, Whyte H, Raybaud C. MR imaging for diagnostic evaluation of encephalopathy in the newborn. Radiographics. 2010 5;30(3):763–780. 10.1148/rg.303095126 20462993

[22] Cowan F, Rutherford M, Groenendaal F, et al. Origin and timing of brain lesions in term infants with neonatal encephalopathy. Lancet. 2003 3 1;361(9359):736–742. 10.1016/S0140-6736(03)12658-X 12620738

